# Na^+^, K^+^-ATPase α Isoforms and Endogenous Cardiac Steroids in Prefrontal Cortex of Bipolar Patients and Controls

**DOI:** 10.3390/ijms21165912

**Published:** 2020-08-17

**Authors:** Shiv Vardan Singh, Olga V. Fedorova, Wen Wei, Haim Rosen, Noa Horesh, Asher Ilani, David Lichtstein

**Affiliations:** 1 The Institute for Medical Research, Israel-Canada, Department of Medical Neurobiology, Faculty of Medicine, The Hebrew University-Hadassah Medical School, Jerusalem 9112102, Israel; vardanshiva@gmail.com (S.V.S.); noa.rosenthal1@mail.huji.ac.il (N.H.); Asheri@ekmd.huji.ac.il (A.I.); 2 Laboratory of Cardiovascular Science, National Institute on Aging, NIH, Baltimore, MD 21224, USA; fedorovo@grc.nia.nih.gov (O.V.F.); wen.wei@nih.gov (W.W.); 3 The Institute for Medical Research, Israel-Canada, Department of Microbiology and Molecular Genetics, Faculty of Medicine, The Hebrew University-Hadassah Medical School, Jerusalem 9112102, Israel; haimr@ekmd.huji.ac.il

**Keywords:** bipolar disorder, Na^+^, K^+^-ATPase, α isoforms, endogenous cardiac steroids, ouabain, marinobufagenin, human, prefrontal cortex, postmortem

## Abstract

Bipolar disorder is a chronic multifactorial psychiatric illness that affects the mood, cognition, and functioning of about 1–2% of the world’s population. Its biological basis is unknown, and its treatment is unsatisfactory. The α1, α2, and α3 isoforms of the Na^+^, K^+^-ATPase, an essential membrane transporter, are vital for neuronal and glial function. The enzyme and its regulators, endogenous cardiac steroids like ouabain and marinobufagenin, are implicated in neuropsychiatric disorders, bipolar disorder in particular. Here, we address the hypothesis that the α isoforms of the Na^+^, K^+^-ATPase and its regulators are altered in the prefrontal cortex of bipolar disease patients. The α isoforms were determined by Western blot and ouabain and marinobufagenin by specific and sensitive immunoassays. We found that the α2 and α3 isoforms were significantly higher and marinobufagenin levels were significantly lower in the prefrontal cortex of the bipolar disease patients compared with those in the control. A positive correlation was found between the levels of the three α isoforms in all samples and between the α1 isoform and ouabain levels in the controls. These results are in accordance with the notion that the Na^+^, K^+^-ATPase-endogenous cardiac steroids system is involved in bipolar disease and suggest that it may be used as a target for drug development.

## 1. Introduction

Bipolar disorder (BD) is a chronic multifactorial psychiatric illness that affects the mood, cognition, and functioning of about 1–2% of the global population [1]. The illness is characterized by episodes of extreme mood states, mania, and depression, interspaced with periods of euthymia. Symptoms of mania include elevated mood, hyperactivity, racing thoughts, insomnia, irritability, and risky behavior. Depression is associated with symptoms, such as sad mood, poor self-esteem, lethargy, and anhedonia. Despite the availability of a broad range of drugs, the treatment of BD remains inadequate: Some patients do not respond to the treatment and many suffer from frequent relapses [2]. A better understanding of the mechanisms involved in BD could contribute to the development of targeted therapies and is of the utmost importance.

Cardiac steroids (CSs), such as the cardenolides ouabain (OUA) and digoxin, and bufadienolides, such as marinobufagenin (MBG), were originally discovered in plants and amphibian skin and are known for their positive inotropic effect [3]. Endogenous cardiac steroids (ECSs), compounds identical or similar to CSs, are present in human tissues and circulation [4,5]. Although the biosynthetic pathway was not fully elucidated, ECSs seem to be synthetized in the adrenal gland and brain [6,7] and are considered hormones involved in numerous physiological and pathological processes, among them cell growth, salt homeostasis, regulation of blood pressure, and behavior [6,7,8,9]. These steroids affect various neuronal functions, suggesting their role as neurosteroids [10,11].

Na^+^, K^+^-ATPase is a ubiquitous plasma membrane transporter that utilizes the energy generated from ATP hydrolysis to catalyze the exchange of intracellular Na^+^ for extracellular K^+^. This enzymatic activity is essential for the regulation of intracellular osmolarity, pH, and calcium concentration; maintenance of the plasma membrane electric potential; and co-transport of substances across the plasma membrane [12]. Na^+^, K^+^-ATPase is a hetero-oligomer composed of two major polypeptides: The α and β-subunits. The α subunit is responsible for the catalytic activity of the enzyme. Three α-subunit isoforms were described in the brain [13]: The ubiquitous α1 isoform; the α2 isoform, which is expressed predominantly in glial cells [14]; and the α3 isoform, which is localized mainly in neurons and dendritic spines [15,16]. The isoforms have different kinetic properties and affinities and they exhibit species-, tissue-, and cell-specific patterns of expression, thus allowing the fine-tuning of Na^+^, K^+^-ATPase activity [13]. Mutations in the α2 and α3 isoforms were implicated in neurological disease activity [13,17].

The α subunit of Na^+^, K^+^ -ATPase is the only established receptor for CS and ECS. Interaction of the steroids with the Na^+^, K^+^-ATPase results in inhibition of the ion-pumping function and causes the activation of several signal transduction cascades, including mitogen-activated protein kinase; extracellular signal-regulated kinase; proto-oncogene tyrosine-protein kinase (Src); the PI3K/Akt, Ca^++^ signaling, and reactive oxygen species generation pathways [18,19]; and TGF-β signaling [20].

Genetic, biochemical, and behavioral studies implicated the Na^+^, K^+^-ATPase and ECS in BD and other mood disorders: A genetic association was described between BD and single nucleotide polymorphisms in the Na^+^, K^+^-ATPase α subunit gene [21]. Mutations in the Na^+^, K^+^-ATPase α3 isoform elicit an array of neurological phenotypes, including manic-like behavior in mice [22,23]. Abnormalities in Na^+^, K^+^-ATPase activity [24] and alterations in ECS levels [25,26,27] were reported in bipolar individuals. A reduction in brain ECS had a protective effect in depressive-like behavior in rats [26], with concomitant alterations in catecholamine levels in specific brain regions [28]. Furthermore, a reduction in brain ECS also protected against manic-like behavior induced by amphetamine (AMPH) in mice [27], an effect that is associated with protection of the brain from oxidative stress [29].

The prefrontal cortex (PFC), a center for executive and cognitive functions [30], is connected to many other brain regions, especially to the limbic brain areas [31]. Numerous studies implicated neuronal activity in the PFC in both the manic and depressive phases of BD [32]: Reduced glial cell number [33] and decreased cortical thickness [34] were found in the PFC of BD; increased gyrification, a marker of early developmental pathology, was found to be increased in BD patients [35]; and discrete miRNA alterations [36] and a reduced density of GABA-synthesizing enzyme, glutamic acid decarboxylase [37], were observed in the PFC of BD patients.

In view of these observations, we hypothesized that the levels of the α isoforms of the Na^+^, K^+^-ATPase and of ECS in the brain may be altered in BD. To test this hypothesis, we compared the levels of the three α isoforms of the Na^+^, K^+^-ATPase and of endogenous OUA and MBG in the PFC of bipolar patients with their levels in age- and sex-matched controls and evaluated the potential correlations among the different isoforms and the two steroids.

## 2. Results

The study was performed on two cohorts of postmortem brain samples of BD and controls obtained from the Human Brain Collection Core (HBCC) of the National Institute of Mental Health (NIMH) Division of Intramural Programs. In both groups, there was no statistical difference in terms of age, gender, postmortem interval, brain weight, and pH between the BD and control samples (see the materials and methods).

### 2.1. Na^+^, K^+^-ATPase α Isoforms in PFC of BD Patients and Controls

**Figure 1:**
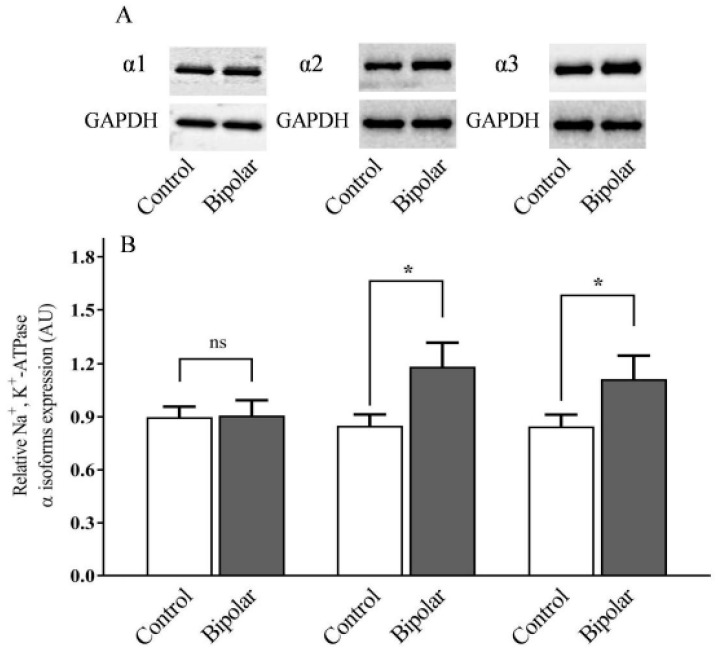
Na^+^, K^+^ ATPase α isoform expression in PFC of BD patients and controls. Na^+^, K^+^-ATPase α subunit isoform expression in postmortem prefrontal cortex samples of controls and BD patients (*n* = 20 per group) was determined by Western blot analysis, as described in the materials and methods. (**A**): Representative Western blots. (**B**): Quantitative isoform expression. The values were normalized to glyceraldehyde 3-phosphate dehydrogenase (GAPDH). The bars represent the means; the error bars represent the standard error of the means; * differ from the control group *p* < 0.05; ns: non-significant.

The levels of the α isoforms of the Na^+^, K^+^-ATPase in the brains of normal and BD patients have been barely investigated. As in a preliminary study on a small cohort of BD patients and controls (six in each group), the levels of the α isoforms were extremely variable (data not shown), we initiated a larger study comparing the α isoform expression levels of 20 BD patients and 20 controls. An example of the Western blots and a quantitative analysis of all the data is shown in Figure 1A,B, respectively.

**Figure 2:**
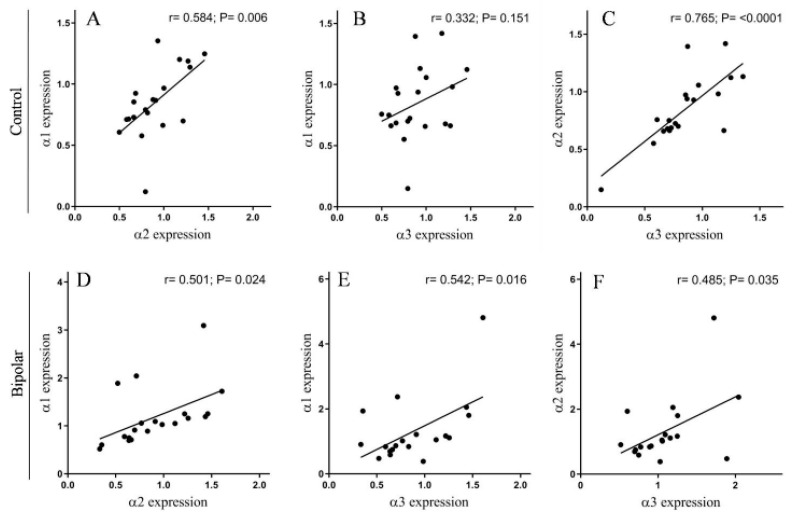
The correlation between the expression of the Na^+^, K^+^-ATPase α isoforms in the PFC of controls and BD patients. The different isoform expression levels (Figure 1) in the controls (**A**–**C**) and BD patients (**D**–**F**) were correlated. The Pearson correlation coefficient (*r*) and the *p* value (two tailed) were calculated by using correlation analysis (GraphPad Prism v 8.3.1).

The Na^+^, K^+^-ATPase α2 and α3 isoforms were significantly higher by 28.2% and 23.7%, respectively, in the PFC of BD patients compared with those in the controls. No difference in α1 isoform abundance between the two groups was detected (Figure 1). The cross-correlations between the levels of expression of the three α isoforms of the Na^+^, K^+^-ATPase were significant in both the controls (Figure 2A–C) and in the BD patients (Figure 2D–F). Namely, α1 was positively correlated with a2 and α3 in the controls (Figure 2A–B) and in the BD patients (Figure 2D–E); the α2 and α3 isoforms were highly correlated in both the controls (Figure 2C) and in the BD patients (Figure 2F).

No positive correlation was found between the levels of any of the three α isoforms and the age of onset of the disease, age of death, or gender of the subjects (data not shown).

### 2.2. Endogenous OUA and MBG in the PFC of the BD Patients and the Controls

Endogenous OUA and MBG are ligands of Na^+^, K^+^-ATPase, which triggers the inhibition of ion transport and intercellular signaling cascades in different tissues, including the brain (see the introduction). We tested, therefore, the levels of these steroids in PFC brain samples from the BD patients and the controls. Importantly, the tissue samples used for the determination of endogenous OUA and MBG were adjacent to the samples used for the protein extraction and the α isoform determinations described above. As the determinations of OUA and MBG in this study were based on the interaction with antibodies, actually, cross-immunoreactive material was being measured. However, because the anti-OUA antibodies and anti-MBG antibodies used were previously shown to be highly specific, recognizing predominantly OUA and MBG, respectively (see the materials and methods), the terms endogenous OUA and endogenous MBG are used.

**Figure 3:**
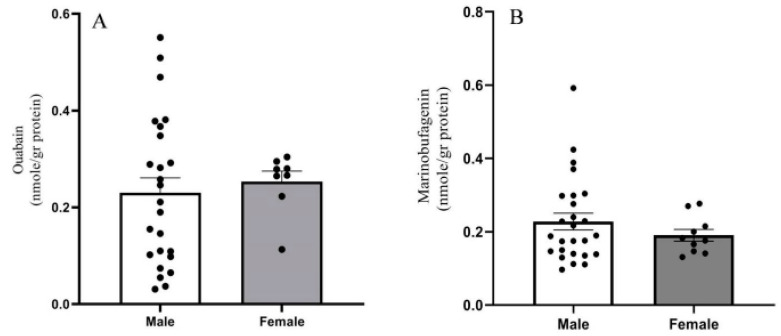
Endogenous OUA- and MBG levels in PFC in men and women. OUA (**A**) and MBG (**B**) immuno-reactivity levels in the postmortem PFC of all samples assayed were determined with DELFIA, as described in the materials and methods. Bars represent the means; error bars represent the standard error of the means.

**Figure 4:**
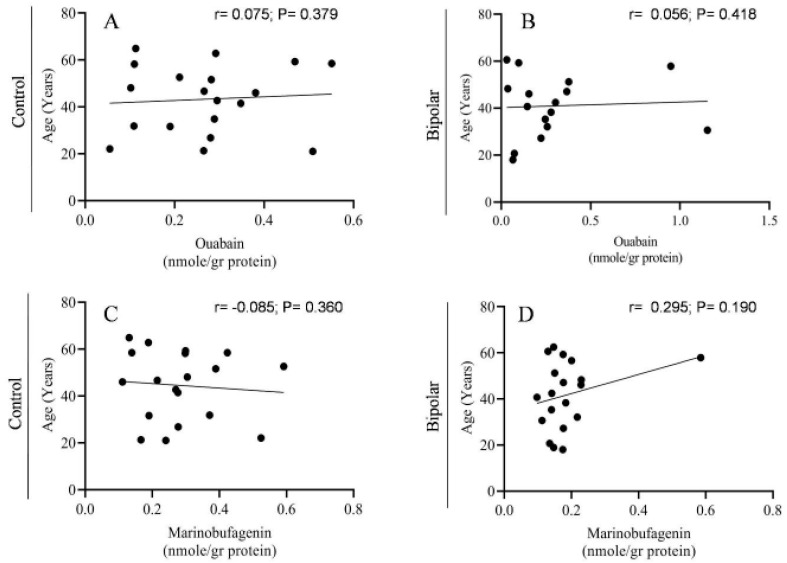
The correlation between endogenous OUA and MBG levels in the PFC with age. The OUA and MBG immuno-reactivity levels of PFC samples of controls (**A**,**C**) and BD patients (**B**,**D**) were correlated with age. Pearson (*r*) and *p* values (two tailed), depicted in the graphs, were calculated by using correlation analysis (GraphPad Prism v 8.3.1).

**Figure 5:**
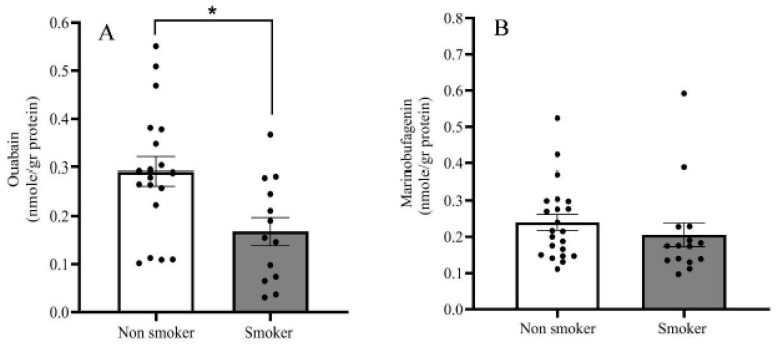
Endogenous OUA and MBG levels in smokers and non-smokers. OUA (**A**) and MBG (**B**) immuno-reactivity levels in the postmortem PFC of all samples assayed were determined by DELFIA, as described in the materials and methods. The bars represent the means; the error bars represent the standard error of the means. * differ from the control group *p* < 0.05.

Both OUA and MBG are present in the human PFC, ranging between 0.1 and 0.5 nmoles/g protein (or 0.3–1.5 pmoles/g tissue). Analysis of all the samples showed no difference in steroid levels between men and women (Figure 3), nor any correlation with age (Figure 4). Significantly lower levels of OUA (0.177 ± 0.028), but not of MBG, were detected in smokers compared with those in non-smokers (0.291 ± 0.0316) (Figure 5).

**Figure 6:**
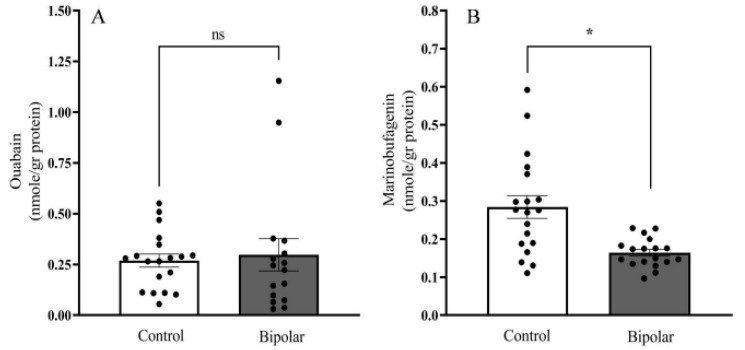
Endogenous OUA- and MBG levels in the PFC of BD patients and controls. OUA and MBG immuno-reactivity levels in postmortem PFC samples of controls (**A**) and BD (**B**) patients (*n* = 16–19) were determined with DELFIA, as described in the materials and methods. The bars represent the means; the error bars represent the standard error of the means; * differ from the control group *p* < 0.05; ns: non-significant.

**Figure 7:**
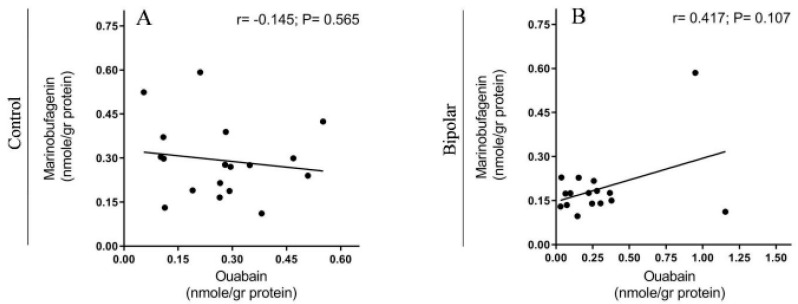
The correlation between endogenous OUA and MBG levels in the PFC of BD patients and controls. The levels of OUA and MBG immuno-reactivity (Figure 3) in BD patients (**B**) and controls (**A**) were correlated. The Pearson correlation coefficient (*r*) and *p* values (two tailed) were calculated by using correlation analysis (GraphPad Prism v 8.3.1).

Significantly lower levels of MBG (34.5%) were detected in BD patients (0.164 ± 0.009 nmoles/g protein) as compared with those in the controls (0.298 ± 0.075 nmoles/g protein) (Figure 6). The OUA levels did not differ between the groups (controls, 0.269 ± 0.032; BD patients, 0.284 ± 0.029 nmoles/g protein). Cross-correlation analysis between the OUA and MBG levels in the controls (Figure 7A) and in the BD patients (Figure 7B) showed a lack of association between the two steroids, suggesting that they differ both metabolically and functionally (see below).

**Figure 8:**
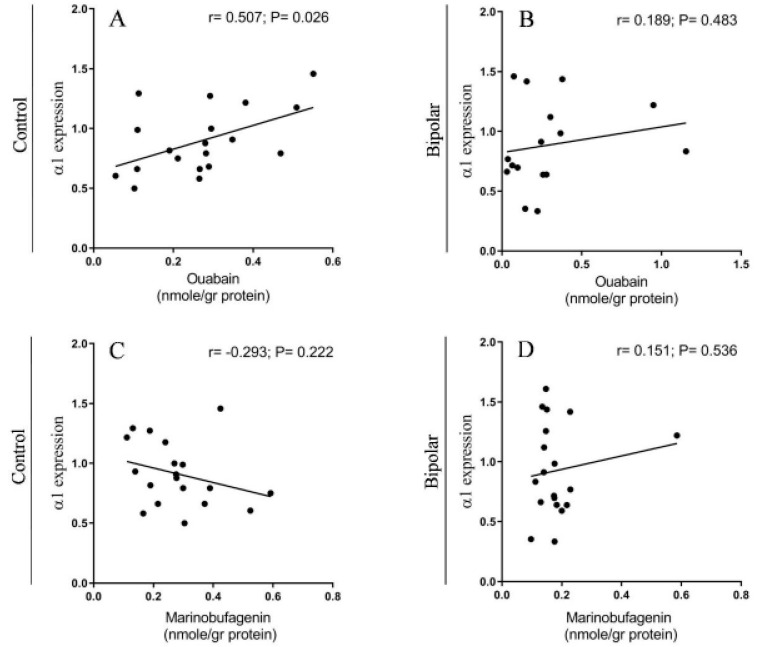
The correlation between the α1 isoform levels and endogenous OUA and MBG immuno-reactivity in the PFC of BD patients and controls. The levels of the α1 isoform of the Na^+^, K^+^-ATPase (Figure 1) and OUA and MBG immuno-reactivity (Figure 3) in BD patients (**C**,**D**) and controls (**A**,**B**) were correlated. The Pearson correlation coefficient (*r*) and the *p* value (two tailed) were calculated by using correlation analysis (GraphPad Prism v 8.3.1).

Cross-correlations between the levels of different α isoforms and the steroids revealed interesting observations. The correlation between the α1 isoform and the OUA levels in the BD patients and the controls is shown in Figure 8A,B. Whereas a significant correlation between the two parameters was seen in the controls (Figure 8A), no correlation was observed in the BD samples (Figure 8B). There was no significant correlation between the α1 isoform and MBG levels in the samples of both the controls and the BD patients (Figure 8C,D). The correlations between the α2 and α3 isoforms and the two steroids in the controls and the BD patients were not significant (see Supplementary Materials file 1, Figure 1 and Figure 2).

## 3. Discussion

In the present study, we found that the levels of the α2 and α3 isoforms of the Na^+^, K^+^-ATPase are significantly higher in the PFC of BD patients in comparison with those of age- and gender-matched controls. This is in agreement with our previous study demonstrating an increase in the α2 and α3 isoforms in the parietal cortex of BD patients [38]. A study on the temporal cortex, however, showed that the levels of the three α isoforms were not significantly different between BD patients and controls [26]. Furthermore, lower α2, but not α1 or α3, isoform levels were found in the temporal cortex of BD patients [39]. Cumulatively, these findings show that the levels of the αisoforms of the Na^+^, K^+^-ATPase in the brain differ between BD patients and controls and that the changes vary between different brain regions. It is well established that alterations in both neuronal [40,41] and glial cells [42,43] occur in the brain of BD patients. It is therefore not surprising that we detected alterations in both the α2 and α3 isoforms, which are largely expressed in glial [14] and neuronal cells [15,16], respectively. The increase in α2 and α3 isoforms in the PFC of BD patients may be part of the etiology of the disease or a consequence of its development. In either case, it is reasonable to suggest that the increase in the isoform levels results from the increased activity of the particular cells. This is similar to the upregulation of muscle a2 isoform levels following exercise [44] and the increase in the α3 isoform following excessive neuronal stimulation [17]. Importantly, since all BD patients received psychoactive drugs (Table 1), we cannot discriminate between the effect of the disease and a possible effect of the medications.

We observed a significant positive correlation between the level of expression of the three α isoforms of the Na^+^, K^+^-ATPase in the PFC of the controls (Figure 2A–C) and BD patients (Figure 2D–F). Namely, individuals with a high α1 isoform level also had a relatively high level of the α2 and α3 isoforms. This link in the expression of the isoforms suggests the existence of a regulatory relationship between the three isoforms. Indeed, it was previously demonstrated that knockdown of one isoform affects the expression of the others. For example, knockdown of the α2 isoform in skeletal muscle cells upregulated the α1 isoform 2.5-fold [45]. In addition, an increase in α1-mRNA decreased α3-mRNA levels in the aging rat cerebral cortex [46], but α1 and α3 changed in an opposite way in heart failure, as did α1 and α2 in heart hypertrophy [47]. In view of the positive relationships between the isoforms in the brain tissue of the controls and the BD patients, it is reasonable to assume that when brain Na^+^, K^+^-ATPase levels increase or decrease, in different people, due to general metabolic changes, they will be reflected by similar changes in the three α isoforms. Such fluctuations may result, for example, from cerebral ischemia or lipid metabolism, which are known to alter Na^+^, K^+^-ATPase expression [48,49,50].

ECSs are normal constituents of the bovine hypothalamus, rat brain, and human CSF [11]. Immunohistochemical studies of mammalian brains revealed high concentrations of these steroids in the paraventricular nucleus and the supraoptic nucleus [51]. Cultured rat hypothalamic neurons were shown to secrete CS in vitro [51,52], supporting the premise that the hypothalamus is the source of endogenous brain CS. The physiological role of ECS in the brain and periphery was recently reviewed [8,11]

We did not observe any difference in OUA and MBG levels in the PFC between men and women nor any correlation with age (Figure 3 and Figure 4). However, there were lower levels of OUA in the PFC of smokers compared with those in non-smokers (Figure 5). A previous study showed increased levels of plasma OUA in men versus women, and in smokers versus non-smokers [53,54]. These differences between changes in the brain and peripheral OUA levels further emphasize the metabolic separation of different ECS in the two compartments, as described previously [8].

The determination of ECS in the brain tissue of BD patients may shed light on the possible involvement of these steroids in this pathological state. In a previous study on the parietal cortex, we found that OUA, but not MBG, levels were significantly higher in BD patients than in normal individuals [26]. In the temporal cortex, opposite results were obtained: Endogenous OUA levels were lower in BD patients relative to that of the controls. However, these difference were not of statistical significance, probably due to the small group studied and the large variations in endogenous OUA levels in the population [38]. To resolve this contradiction, and to focus on a brain area more relevant to BD, the present study determined OUA and MBG levels in larger groups (20 samples per group) of postmortem samples from the PFC of BD and normal subjects. Our results show that the levels of MBG in the PFC of BD patients are lower than those in normal subjects (Figure 6). OUA levels, on the other hand, were the same in the two groups. Clearly, these findings indicate that MBG may be involved in the etiology of BD. In addition, the reported effects of smoking on Na^+^, K^+^-ATPase activity [55] and the recently described finding of a causal risk factor of smoking for developing bipolar disorder [56] should prompt a study on the determination of brain OUA and MBG levels in a large group of BD and controls.

The lack of a correlation between OUA and MBG in both the controls and the BD patients (Figure 7) suggests that the two compounds, although structurally similar, are metabolically separated. Indeed, previous studies showed that although cholesterol is a common precursor for both steroids, side-chain cleavage is essential for EO biosynthesis [8,57], whereas MBG synthesis is thought to occur via further metabolism of cholanic acid [58]. The differences in the levels of the steroids in BD are in accord with recent observations of altered steroid biosynthesis in the PFC of BD patients [59].

A significant positive correlation was found between the OUA and α1 isoform levels in the PFC of the control (Figure 8A). The most plausible explanation for this correlation is that OUA has a regulatory role in controlling the expression of this isoform. The many observations on the effect of OUA on the translation and transcription of numerous proteins [60,61] are in accord with this notion. It should be noted that this correlation is absent in samples from BD patients (Figure 8B), suggesting that fundamental metabolic processes related to the ECS-Na^+^, K^+^-ATPase system are impaired in the disease state.

The hypothesis that monoamine depletion contributes to mood disorder pathology, a notion referred to as the ‘monoamine hypothesis’, received great attention in neurobiological studies of mood disorders [62]. Accordingly, monoamine neuronal reuptake and degradation inhibitors were developed for the treatment of mood disorders. However, the slow pace of action of these drugs, their side-effects, and poor response in a significant proportion of patients suggests that additional mechanisms participate in the pathophysiology of mood disorders and of BD in particular. To this end, studies in recent years have focused on the involvement of additional neurotransmitter/neuromodulators systems [63,64,65], mitochondrial function [66], and inflammation [67] in the etiology of BD. It is well established that Na^+^, K^+^-ATPase and ECS affect all these systems: They are involved in neurotransmitter release and reuptake [10,68], mitochondrial function [69], and inflammation [70]. Hence, the increase in the α2 and α3 isoforms of the Na^+^, K^+^-ATPase and the decrease in MBG in the PFC of BD patients, as found in this study, may be an intimate part of the molecular mechanisms of BD. Whether these changes are a cause or consequence of BD merits further investigation.

The present study describes for the first time alterations in Na^+^, K^+^-ATPase α isoforms and endogenous OUA and MBG in the PFC of BD patients., Our results, together with previously published observations, are in accord with the hypothesis that the Na^+^, K^+^-ATPase-ECS system is involved in mood disorders.

**Figure 9:**
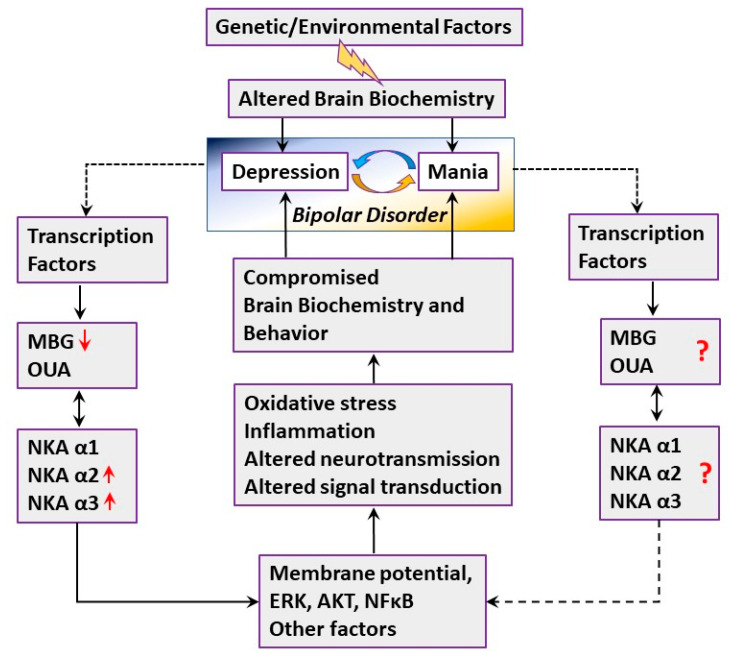
Proposed model for the participation of Na^+^, K^+^-ATPase and endogenous cardiac steroids in bipolar disorders. See text for detail. NKA-Na^+^, K^+^-ATPase; OUA—Ouabain; MBG—Marinobufagenin; ERK—mitogen-activated protein kinase; AKT—protein kinase B; NFkB—nuclear factor kappa-light-chain-enhancer.

The proposed mechanisms for the participation of Na^+^, K^+^-ATPase and ECS in BD are depicted in Figure 9. We suggest that the bipolar brain, being predisposed to the disease via an altered genome and biochemistry, exhibits the manic or depression reaction as a result of external stimuli, which include genetic and environmental factors. The death of most of the BD patients in the present study was attributed to suicide (Table 1), which suggests that they were likely in the depression state. It is possible that neurons and other brain cells stimulate multiple factors, including transcription factors, which affect brain ECS production in the depression state. These transcription factors should be determined (dotted line). We observed a decrease in the brain MBG level (Figure 6), which is likely due to the compromised biosynthesis chain of this steroid. It is conceivable that decreased MBG levels affected Na^+^, K^+^-ATPase activity, causing an increase in the levels of α2 and α3 Na^+^, K^+^-ATPase isoforms (Figure 1). The double arrow between the CSs and Na^+^, K^+^-ATPase boxes indicates the feedback in the regulation of the ECS level and Na^+^, K^+^-ATPase isoforms’ expression. The Na^+^, K^+^-ATPase–ECS interaction leads to changes in the membrane electrical potential, and the activation of mitogen-activated protein kinase (ERK, or MAPK), protein kinase B (AKT), and nuclear factor kappa-light-chain-enhancer (NFκB) [19,71], which modifies neuronal activity and neurotransmission that, in turn, participate in the regulation of behavior and BD. The oxidative stress and inflammation, which are also involved in the Na^+^, K^+^-ATPase–ECS interaction [6,29], may add to the compromised brain biochemistry and support the vicious circle of depression in BD. We also suggest that the levels of ECS are changed in the mania stage, which may cause the compromised interaction of ECS and Na^+^, K^+^-ATPase. The mania circle requires further investigation. The determination of ECS levels in the plasma or cerebrospinal fluid of BD patients may provide an additional mechanistic basis for the proposed mechanisms involved in the two stages of BD.

## 4. Materials and Methods

### 4.1. Brain Samples

All postmortem human brain tissue samples used in this study were obtained from the Human Brain Collection Core (HBCC), Intramural Research Program, of the NIMH, NIH, Bethesda, MD, USA [72]. Two groups of samples were received: 12 prefrontal brain samples (6 BD and 6 controls), which were used in a preliminary study, and a large cohort of prefrontal brain samples (20 BD and 20 controls). The demographic and clinical characteristics of the large cohort are shown in Table 1. The two groups were matched for several clinical variables. According to analysis of variance (*ANOVA*), the groups did not differ in age or postmortem interval, brain weight, and pH. Chi square analysis indicated that the groups did not differ in terms of gender. The NIMH received ethics approval for the brain collection.

Pulverized frozen tissues of the PFC of the two groups were used for the determination of the different α isoforms with Western blot analysis and for the determination of the endogenous OUA and MBG.

### 4.2. Quantification of Na^+^, K^+^-ATPase α Isoforms with Western Blot

Pulverized frozen tissues of the prefrontal cortex were kept at −80°C until analyzed. Samples were homogenized in radio-immuno-precipitation assay (RIPA) buffer supplemented with 1mM NaVO_4_ and Protease Inhibitor Cocktail (Sigma-Aldrich, St. Louis, MO, USA) and centrifuged (14,000× *g*). The protein content of the supernatants was determined with the Bio-Rad Protein Assay (Bio-Rad Laboratories, Hercules, CA, USA) according to the manufacturer’s instructions.

The samples were subjected to Western blot analysis, as previously described [26]. The following primary antibodies were used: mouse monoclonal anti-Na^+^, K^+^-ATPase-α1 subunit antibody (1:10000) (Merck, Kenilworth, NJ, USA); rabbit polyclonal anti-Na^+^, K^+^-ATPase-α2 subunit antibody (1:5000) was kindly provided by Thomas Pressley (Texas Tech University, Lubbock, TX, USA); mouse monoclonal anti-Na^+^, K^+^-ATPase-α3 subunit antibody (1:5000) and mouse monoclonal anti-GAPDH antibody were purchased from Sigma-Aldrich. Western blot analysis was performed by an individual blinded to the subjects’ identity.

### 4.3. Determination of Endogenous OUA and MBG in Brain Samples

Human PFC samples were thawed and homogenized in PBS (50 mg/mL), sonicated for 5 s, and centrifuged (5 min, 1000 × g) to remove tissue debris. The supernatants were used for the protein measurements (Bio-Rad Protein Assay) and for steroid extraction with C18 Sep-Pak cartridges (Waters Inc., Cambridge, MA, USA). The cartridges were activated with 10 mL of 100% acetonitrile and washed with 10 mL of water. Then, 0.5 mL of brain extract sample were applied to the cartridges and eluted with 7 mL of 20% acetonitrile, followed by 7 mL of 80% acetonitrile in the same vial. This enabled the elution of material with lower and higher polarity, respectively, and allowed measurement of the OUA and MBG in the samples [73]. The samples were then vacuum dried and kept at −80°C. Before the immunoassays, the samples were reconstituted in the initial volume of assay buffer. MBG and OUA were measured with a fluoroimmunoassay (Dissociation-Enhanced Lanthanide Fluorescent Immunoassay (DELFIA)). The MBG assay is based on a murine monoclonal anti-MBG 4G4 mAb (1:1000), as described in detail [74]. This assay is based on the competition between immobilized antigen (MBG-bovine serum albumin (MBG-BSA) glycoside-thyroglobulin) and MBG, other cross-reactants, or endogenous CTS within the sample for a limited number of binding sites on an anti-MBG mAbs. Secondary (goat anti-mouse) antibody labeled with fluorescent europium was obtained from Perkin-Elmer, Inc. (Waltham, MA, USA). More details about these immunoassays, including cross-immunoreactivity of the antibodies and the examples of the calibration curves, are provided in the (Supplementary Material file 2). The endogenous OUA assays were based on a similar principle, here using an ouabain–thyroglobulin conjugate and ouabain antiserum (anti-OU-M, 1:20000) obtained from rabbits immunized with a mixture of ouabain–BSA and ouabain–RNAase conjugates [74]. Secondary (mouse anti-rabbit) antibody labeled with fluorescent europium was obtained from Perkin-Elmer, Inc. The final concentration of the endogenous steroids in the PFC was expressed as nmoles/g protein and in pmoles/g protein. The calculation was based on the concentration of steroids and concentration of protein in the supernatant and/or the amount of tissue in grams used for the extraction. Determination of the steroids was performed by an individual blinded to the subjects’ identity.

### 4.4. Statistical Analysis

The data are presented as the means ± standard error. The band density values obtained from the immunostained α isoforms and ECS were analyzed statistically with the two-tailed paired t-test. Parameter levels ±3S.D. from the mean were considered outliers (maximum 3 samples out of the 20) and deleted from statistical analysis. Statistical analysis and calculations of Pearson’s correlation coefficient were performed with GraphPad Prism v 8.3.1 (GraphPad Software, Inc., CA, USA) and Gaussian Population (Pearson) with two-tailed parametric analyses *p* < 0.05 was considered significant.

## Figures and Tables

**Table 1:** Characteristics of BD patients and unaffected controls.

	Control (*n* = 20)	Bipolar Patients (*n* = 20)
Age at death (years)	43.12 ± 3.21	42.25 ± 3.23
Cause of death (Suicide/other)	0/20	15/5
Brain weight (g)	1382.75 ± 23.08	1511.75 ± 37.58
Postmortem interval (h)	30.70 ± 3.03	28.97 ± 3.94
Brain tissue pH	6.44 ± 0.05	6.34 ± 0.05
Sex (Men/Women)	14/6	14/6
Alcohol history (none/positive)	4/16	5/15
Use of Psychoactive drugs (none/positive)	14/6	0/20

## Supplementary Materials

The following are available online at https://www.mdpi.com/1422-0067/21/16/5912/s1. Supplementary file 1 contains Supplementary Figures. Supplementary file 2 contains additional information on material and methods.

## Abbreviations

BD:	Bipolar Disorder
PFC:	Prefrontal Cortex
CS:	Cardiac Steroids
ECS:	Endogenous Cardiac Steroids
OUA:	Ouabain
MBG:	Marinobufagenin
Na^+^, K^+^-ATPase:	Sodium-Potassium-Activated Adenosine Triphosphatase
HBCC	Human Brain Collection Core
