# Supplementary material for: Na+, K+-ATPase α Isoforms and Endogenous Cardiac Steroids in Prefrontal Cortex of Bipolar Patients and Controls
Source: Int J Mol Sci. 2020 Aug 17;21(16):5912. doi: 10.3390/ijms21165912 (PMC7460572; doi:10.3390/ijms21165912)
Supplement: Supplementary file 1 [file ijms-21-05912-s001.zip › Supplement-2.docx]

**Supplement 2**

**Cross-immunoreactivity of anti-OUA and anti-MBG antibodies used in immunoassays**

For our competitive reverse phase immunoassays, we used highly specific anti-MBG monoclonal antibody (mAb; clone 4G4) or polyclonal anti-OUA antibody (Methods, 4.3). Several steroids, including cardenolides and bufadienolides, were used to study the immunoreactivity of anti-OUA-M and anti-MBG antibodies. Steroids were diluted in the assay buffer and tested in our immunoassays to compete with immobilized antigen (MBG-BSA) for a limited number of binding sites on anti-MBG mAb (clone 4G4) or with immobilized antigen OUA-thyroglobulin for a limited number of binding sites on anti-OUA-M polyclonal antibody (Table 1). Anti-MBG mAb demonstrated very low cross-immunoreactivity with cardenolides, aldosterone, prednisone, progesterone and corticosterone. Bufadienolides, i.e., cinobufotalin, marinobuftoxin and telocinobufagin, have high cross-reactivity with anti-MBG mAb, due to close chemical structure to MBG. Anti-OUA antibody exhibited low cross-reactivity with all studied steroids except ouabagenin. Notably, the cross-reactivity of anti-MBG antibody with OUA is very low (0.005%), and vise-versa, cross-reactivity of anti-OUA antibody with MBG is 0.15%, which indicate that our immunoassays uniquely recognized the correspondent steroids in the brain samples. The specificity of our immunoassays is also supported by the absence of correlation between brain endogenous MBG and OUA in both BD and controls (Figure 7).

**Table 1.** *Cross-immunoreactivity of anti-MBG mAb and anti-OUA-M polyclonal antibody with different steroids*

| Cross-reactants (steroids): | Cross-reactivity with 4G4 anti-MBG monoclonal antibody (%) | Cross-reactivity with anti-OUA polyclonal antibody (%) |
| --- | --- | --- |
| MBG | **100** | 0.15 |
| Marinobufotoxin | 43 | 0.2 |
| Cinobufotalin | 40 | 0.1 |
| Telocinobufagin | 14 | 1.2 |
| Resibufagenin | 0.5 | 0.03 |
| Bufalin | 0.08 | 0.23 |
| Cinobufagin | 0.07 | n/d |
| Digoxin | 0.03 | 1.5 |
| Ouabain | 0.005 | **100** |
| Ouabagenin | 0.001 | 21 |
| Proscillaridin A | <0.001 | 0.09 |
| Digitoxin | <0.001 | 0.8 |
| Aldosterone | <0.001 | 0.01 |
| Progesterone | <0.001 | 0.02 |
| Prednisone | <0.001 | 0.004 |
| Corticosterone | <0.001 | 0.001 |

**MBG and OUA measurement.** The detailed description of the fluoroimmunoassays for MBG and OUS measurements is given in the Methods section (4.3). Standard calibration curves for the MBG immunoassay and for OUA immunoassay depicted below. The concentrations of OUA and MBG in the brain samples are added to the graphs in color symbols. Concentrations of both OUA and MBG in the brain samples were detected in the diapason between 10^-10^ and 10^-9^ moles/L.

**Figure 1.** *Displacement of binding* of anti-OUA-M polyclonal antibody to OUA-Tg conjugate by OUA standard (black) and unknown samples (blue) in competitive reverse phase immunoassay (A). Displacement of binding of 4G4 anti-MBG monoclonal antibody to MBG-BSA conjugate by MBG standard (black) and unknown samples (red) in competitive reverse phase immunoassay (B). MBG, marinobufagenin; OUA, ouabain.
