# Supplementary material for: Na+, K+-ATPase α Isoforms and Endogenous Cardiac Steroids in Prefrontal Cortex of Bipolar Patients and Controls
Source: Int J Mol Sci. 2020 Aug 17;21(16):5912. doi: 10.3390/ijms21165912 (PMC7460572; doi:10.3390/ijms21165912)
Supplement: Supplementary file 1 [file ijms-21-05912-s001.zip › Supplement-1.docx]

**Supplement 1**

**Figure 1.** *Correlations between α2 and α3 isoforms of the Na^+^, K^+^-ATPase and the levels of OUA in PFC of control and BD patients*


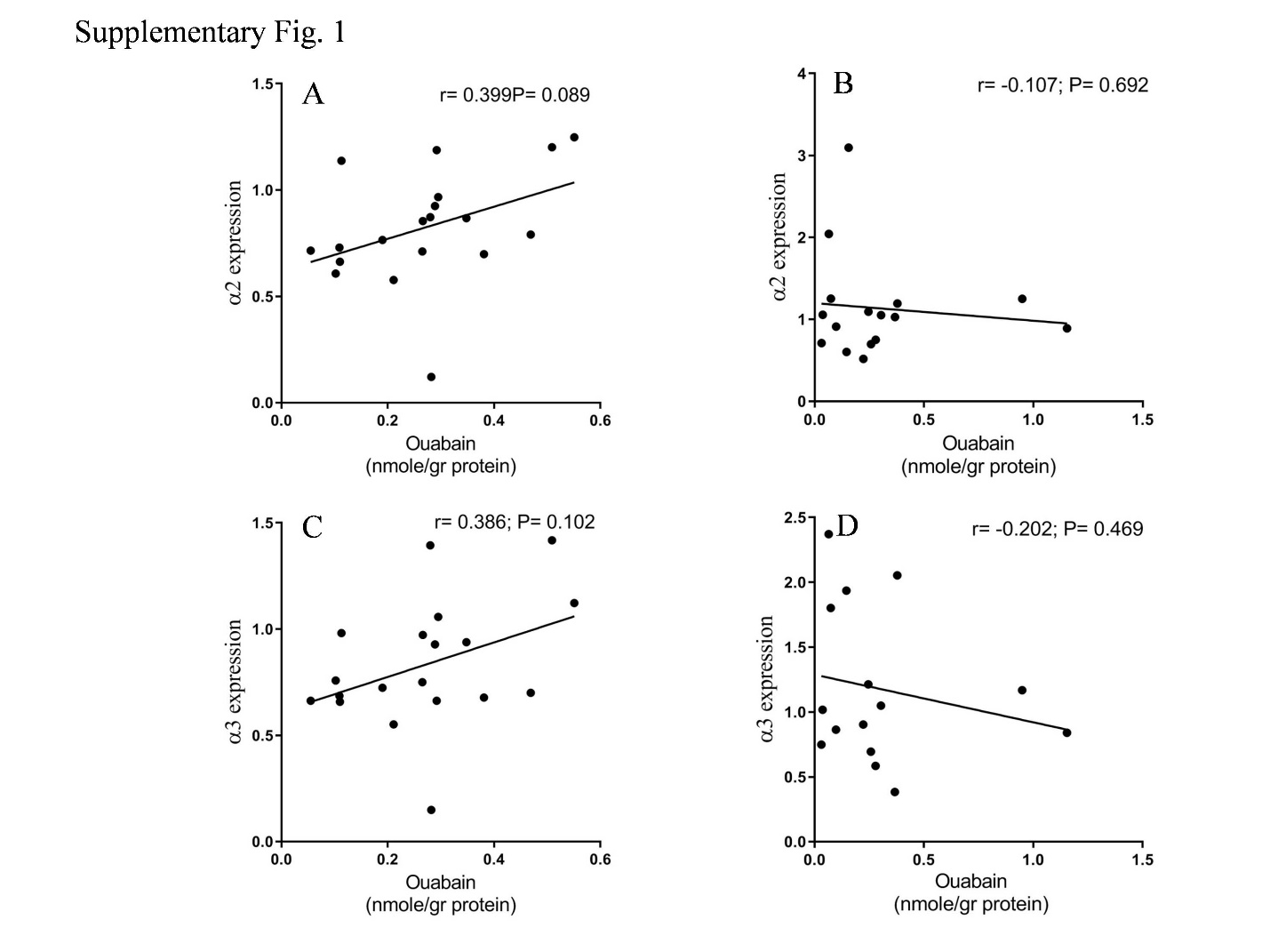


Values of α2 and α3 isoform of the Na^+^, K^+^-ATPase expression (Figure 1) and OUA (Figure 6) in control (A and C) and BD patients (B and D) were correlated. Pearson (r) and P value (two tailed), depicted in the graphs, were calculated using correlation analysis (GraphPad Prism v 8.3.1).

**Figure 2.** *Correlations between a2 and a3 isoforms of the Na^+^, K^+^-ATPase and the levels of MBG in PFC of control and BD patients.*


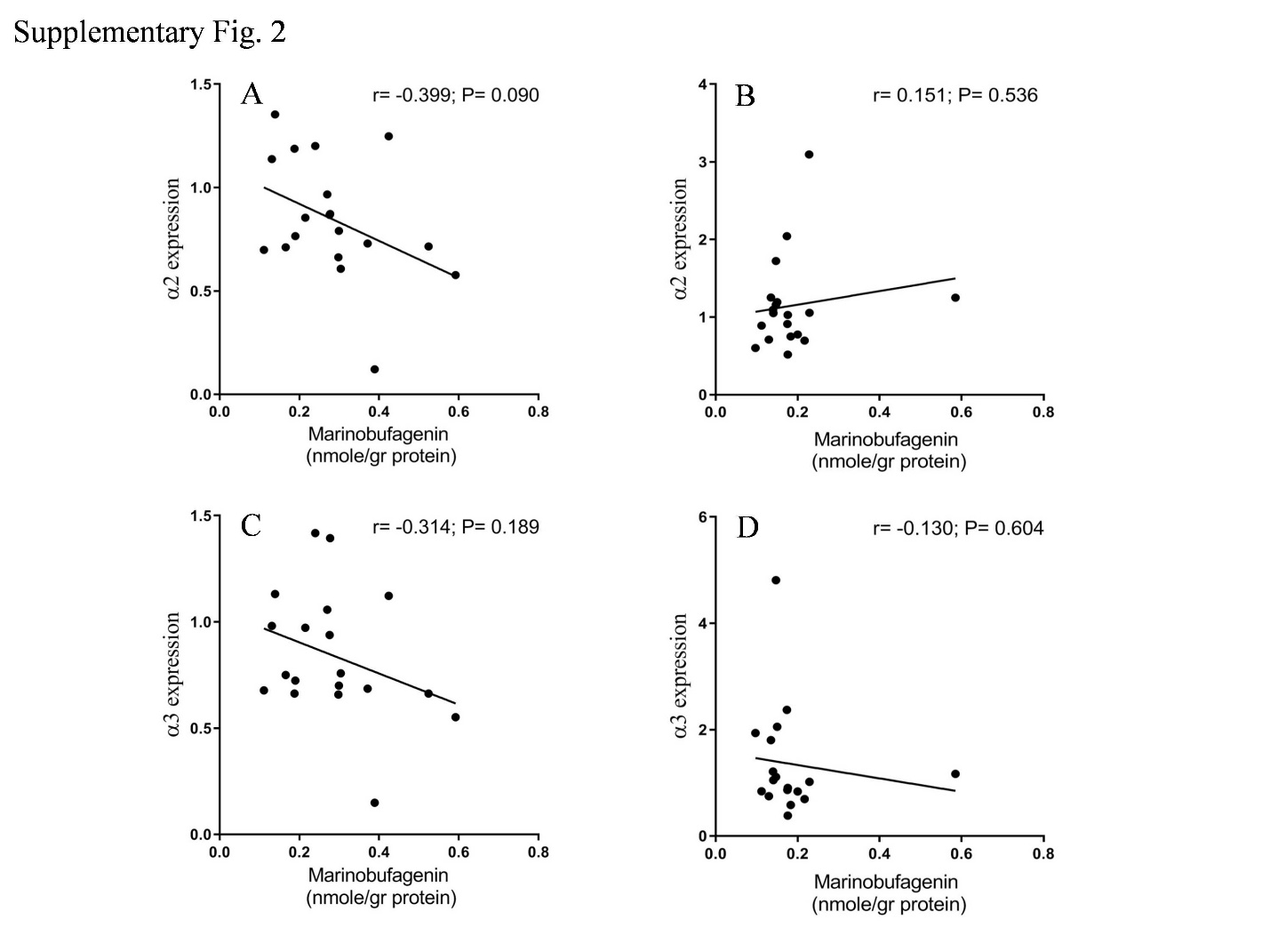


Values of α2 and α3 isoform of the Na^+^, K^+^-ATPase expression (Figure 1) and MBG (Figure 6) in control (A and C) and BD patients (B and D) were correlated. Pearson (r) and P value (two tailed), depicted in the graphs, were calculated using correlation analysis (GraphPad Prism v 8.3.1).
